# PlantMine: A Machine-Learning Framework to Detect Core SNPs in Rice Genomics

**DOI:** 10.3390/genes15050603

**Published:** 2024-05-09

**Authors:** Kai Tong, Xiaojing Chen, Shen Yan, Liangli Dai, Yuxue Liao, Zhaoling Li, Ting Wang

**Affiliations:** 1 School of Biological Engineering, Sichuan University of Science & Engineering, Yibin 644000, China; tongkai@suse.edu.cn (K.T.); 322086002309@stu.suse.edu.cn (L.D.); 323083202108@stu.suse.edu.cn (Y.L.); 2 National Agriculture Science Data Center, Agricultural Information Institute, Chinese Academy of Agricultural Sciences, Beijing 100081, China; 82101225580@caas.cn; 3 National Nanfan Research Institute, Chinese Academy of Agricultural Sciences, Sanya 572024, China; 4 State Key Laboratory of Crop Gene Resources and Breeding, Institute of Crop Sciences, Chinese Academy of Agricultural Sciences, Beijing 100081, China; yanshen@caas.cn; 5 Agricultural Information Institute, Chinese Academy of Agricultural Sciences, Beijing 100081, China; 6 Key Laboratory of Big Agri-Data, Ministry of Agriculture and Rural Areas, Beijing 100081, China

**Keywords:** feature selection, genomic prediction, machine learning, rice breeding, SNP

## Abstract

As a fundamental global staple crop, rice plays a pivotal role in human nutrition and agricultural production systems. However, its complex genetic architecture and extensive trait variability pose challenges for breeders and researchers in optimizing yield and quality. Particularly to expedite breeding methods like genomic selection, isolating core SNPs related to target traits from genome-wide data reduces irrelevant mutation noise, enhancing computational precision and efficiency. Thus, exploring efficient computational approaches to mine core SNPs is of great importance. This study introduces PlantMine, an innovative computational framework that integrates feature selection and machine learning techniques to effectively identify core SNPs critical for the improvement of rice traits. Utilizing the dataset from the 3000 Rice Genomes Project, we applied different algorithms for analysis. The findings underscore the effectiveness of combining feature selection with machine learning in accurately identifying core SNPs, offering a promising avenue to expedite rice breeding efforts and improve crop productivity and resilience to stress.

## 1. Introduction

The quest for global food security is one of the most pressing challenges of the 21st century, exacerbated by a burgeoning global population projected to reach nearly 10 billion by 2050 [1,2] and the multifaceted threats posed by climate change. Rice, as a staple food for over half of the world’s population, is at the heart of this challenge, necessitating innovative approaches to enhance its yield, nutritional value, and resilience to environmental stresses [3]. The genetic improvement of rice through breeding has historically played a pivotal role in addressing food security, with the Green Revolution serving as a landmark achievement that significantly increased crop yields. However, traditional breeding methods are increasingly seen as insufficient to meet the contemporary demands for rapid, sustainable, and environmentally friendly crop improvement [4].

Recent developments in genomics and biotechnology have paved the way for advancements in crop improvement, and breeders are now able to conduct research on the relationship between single nucleotide polymorphisms (SNPs) and various crop phenotypes at the genome-wide level. As the most prevalent form of genetic variation, genome-wide SNPs are essential for dissecting the genetic foundations of key phenotypic traits such as yield, disease resistance, and drought tolerance [5,6,7]. Leveraging the analysis and processing of genome-wide SNPs in rice breeding can markedly hasten the creation of superior varieties, thereby presenting an effective strategy to bolster food security. Nevertheless, the challenge of handling SNPs at the genome-wide level lies in their high dimensionality and sheer volume. Utilizing unselected and unfiltered SNPs directly in subsequent analyses can lead to significant statistical challenges, a situation exacerbated by the small sample sizes available in current rice genomic datasets. Consequently, it is crucial to choose appropriate feature extraction methods to isolate important SNPs closely associated with target phenotypes [8], known as core SNPs, from the multitude of genome-wide SNPs. This selection aids in supporting further research on genomic prediction and related studies, ensuring more targeted and effective analyses. Various methods for identifying core SNPs have been developed. These methods rely on manual identification, use tools such as Genome-Wide Association Studies (GWAS), or focus solely on the diversity between genotypes, disregarding the intended phenotypes for computation [9,10]. However, these methods have various shortcomings. For example, methods based on manual identification are time-consuming and costly, methods using GWAS lack the ability to capture minor and rare mutations, and methods that focus only on genotypes to maximize genetic diversity struggle to retain sufficient information for targeted phenotypes. Most importantly, these methods only focus on the single step of capturing core SNPs and cannot meet the needs of subsequent algorithms for predicting different phenotypes. Machine learning (ML) has revolutionized data analysis by providing sophisticated tools to handle the vast and complex datasets generated by recent genomic studies. ML techniques, particularly feature selection methods, have shown great potential for identifying the most informative genetic markers from large sets of SNPs, thereby improving the efficiency and accuracy of genomic selection [11]. Feature selection not only aids in reducing the dimensionality of genomic data but also enhances model interpretability and reduces overfitting, making it an invaluable component of ML-based genomic selection strategies [12,13]. Machine learning methods, such as Random Forest, have been utilized for SNPs screening to enhance model predictive capability [14]. Therefore, using appropriate feature selection methods can quickly eliminate the impact of unnecessary features from genome-wide markers in rice, thereby enhancing the accuracy of subsequent analyses and increasing computational speed [15,16]. However, considering the varying compatibility between different feature selection methods and genomic prediction algorithms, it becomes a challenge for breeders to select the most suitable feature selection method based on their genomic prediction algorithms.

**Figure 1:**
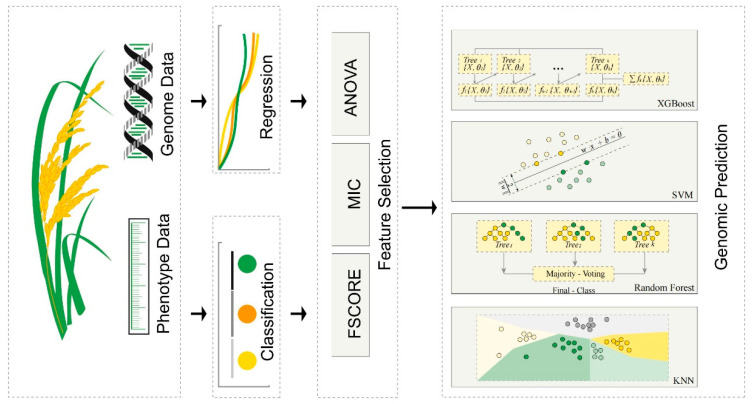
PlantMine framework for detection of core SNPs in rice genomics. The workflow for constructing PlantMine. In this framework, three feature selection methods and four machine learning methods are combined to seek the optimal results. The meanings of the abbreviations in the figure are as follows: ANOVA: Analysis of Variance, MIC: Maximal Information Coefficient, F-SCORE: Fisher Score, XGBoost: eXtreme Gradient Boosting, SVM: Support Vector Machine, KNN: K-Nearest-Neighbors, RF: Random Forest.

To facilitate fast and efficient identification of key SNP sets associated with specific traits, this study introduced an innovative computational framework, PlantMine, which uses three feature selections paired with four machine learning approach techniques, respectively, so as to facilitate the management of high-dimensional feature data and to compare the combinations of techniques that can be used to efficiently mine the core set of SNPs associated with rice breeding, as illustrated in Figure 1. We validated the effectiveness of the PlantMine framework using the phenotype data—day to heading from the rice 3 k dataset as a feature. Utilizing PlantMine, we are able to swiftly determine the optimal feature selection and machine learning-based predictive methodologies based on genotypic and targeted phenotypic data, thereby facilitating the rapid identification of key genetic markers associated with the desired crop phenotypes. This study provides new data analysis and processing approaches for subsequent rice genomic prediction models and whole-genome selection breeding.

## 2. Materials and Methods

### 2.1. Dataset

The 3000 Rice Genomes Project constitutes a comprehensive gigabyte-scale dataset encompassing genome sequences from 3010 distinct rice varieties, capturing the extensive genetic and functional diversity of rice on a global scale [17]. From the rice dataset, we retrieved 2799 samples containing 404,000 core SNPs and the phenotypes data—day to heading from the public database (https://snpseek.irri.org/_download.zul, accessed on 15 December 2023). It should be noted that the 404,000 core SNPs here were initially selected by the provider of the dataset without any regard for the target phenotype. For these SNPs, we first applied a filtering process using PLINK based on linkage disequilibrium (LD) with parameters (indep-pairwise 1000 100 0.1), which resulted in 7202 SNPs. We then conducted a secondary screening of these 7202 SNPs using the PlantMine framework to validate its effectiveness. Considering the differences in quantity and quality traits, we segmented the original continuous data into categorical data. We sorted all the specific heading days data, and regardless of the exact number of days, the shortest 25% were uniformly assigned as Level 1, the middle 25% to 75% were assigned as Level 2, and the longest 25% were assigned as Level 3 [18] (Figure S1, Supplementary S2). These samples were divided into a training set consisting of 2239 samples and a test set consisting of 560 samples, which ensured a robust framework for subsequent analysis and model training.

### 2.2. PlantMine Framework

The PlantMine computational framework establishes two tasks, regression and classification (Figure 1). In the regression task, the goal is to identify SNPs suitable for quantitative trait screening, whereas in the classification task, we classified the genomic variation dataset into three categories of samples based on flowering time, namely, early, mid, and late samples for training, and used them to identify SNPs that are suitable for qualitative trait screening. In order to effectively eliminate useless features and reduce the dimensionality of input features, PlantMine employs three feature selection methods: analysis of variance (ANOVA), maximal information coefficient (MIC), and Fisher score (F-SCORE), which help to improve the operational efficiency of the algorithm. After the feature selection is completed, PlantMine pairs the three feature selection methods with eXtreme Gradient Boosting (XGBoost), Support Vector Machine (SVM), K-Nearest-Neighbors (KNN) and Random Forest (RF), and at the same time, the incremental feature selection (IFS) strategy was adopted to gradually increase the features to select the best subset of features, avoiding the computational complexity and overfitting problems that may be caused by selecting all the features at one time, and ultimately completing the efficient prediction of the core SNPs that are closely related to the key traits of rice resistance and yield.

### 2.3. Feature Selection Method

The feature selection methods used in this study include ANOVA, MIC, and F-SCORE. ANOVA, also known as Fisher’s analysis of variance, tests for statistical significance by the ratio of between-group variance to within-group variance, is a filtering method used to capture the linear relationship between each feature and the labels, and is suitable for both regression and classification tasks [19]. MIC is a non-parametric exploratory statistic based on maximum information for identifying and categorizing larger classes of relationships, which can measure the degree of association between two variables, thus filtering out the more influential characteristic variables [20]. F-SCORE is a measure of the ability of features to discriminate between two classes, a technique that selects specific features such that in the same class the feature values are similar and in dissimilar classes the feature values are completely different [21]. We select the above three representative methods to process the dataset separately to eliminate irrelevant features, reduce the dimensionality of the data, and provide effective information input for the next machine learning algorithm to process the task.

### 2.4. Genomic Prediction Method

After determining that the high-dimensional feature data are processed effectively, we assess the quality of the core SNPs identified by different methods based on their performance in genomic prediction tasks. Until now, many machine learning algorithms have appeared for genomic prediction. In this study, four machine learning models, namely, XGBoost, SVM, KNN, and RF, are used in a collaborative feature selection method to construct the PlantMine framework. In order to reduce the risk of overfitting algorithms and to increase the algorithms’ ability to generalize, an incremental feature selection method was adopted for the prediction of key SNPs related to rice breeding.

#### 2.4.1. XGBoost Algorithm

In this study, we employed the XGBoost algorithm, a state-of-the-art machine learning technique renowned for its efficiency, performance, and flexibility in handling complex datasets. XGBoost is an advanced implementation of gradient boosted decision trees designed for speed and performance. It is particularly adept at handling sparse data and has been widely adopted in various domains, including genomics, for its superior predictive capabilities and efficiency in feature selection [22,23].

#### 2.4.2. SVM Algorithm

To address the feature selection challenges inherent in screening core SNPs in rice, we utilized the SVM algorithm. SVM is a supervised machine learning model that is widely recognized for its robustness and effectiveness in binary classification tasks, making it particularly suitable for genomic data analysis [24,25]. The algorithm operates by finding the hyperplane that best separates the data points of different classes in the feature space, maximizing the margin between the closest points of the classes, which are referred to as support vectors.

#### 2.4.3. KNN Algorithm

The KNN algorithm classifies each data point based on the majority vote of its ‘k’ nearest neighbors, with ‘k’ being a user-defined constant. The proximity between data points is typically measured using distance metrics such as Euclidean, Manhattan, or Minkowski distance. For our study, we chose the Euclidean distance due to its intuitive geometric interpretation and effectiveness in high-dimensional spaces like those encountered in SNP datasets.

#### 2.4.4. RF Algorithm

The RF operates by constructing a multitude of decision trees during the training phase and outputting the class, which is the mode of the classes predicted by individual trees for classification tasks. Each tree in the forest is built from a random sample of the training set, and at each node, a subset of features is randomly selected to determine the split. This approach, known as bootstrap aggregating or bagging, coupled with feature randomness, ensures diversity among the trees, thereby enhancing the model’s generalization ability.

### 2.5. Evaluation Metrics

In order to verify the effectiveness of the proposed framework, the quantitative metrics accuracy, recall, precision, and F1score are used to compare the performance of different combinations, and the above four metrics take values in the range of 0 to 1. A high value indicates good performance of the algorithm, which is defined as follows:

(1) $$Accuracy=\frac{TP+TN}{TP+TN+FP+FN}$$

(2) $$Recall=\frac{TP}{TP+FN}$$

(3) $$Precision=\frac{TP}{TP+FP}$$

(4) $$F1score=\frac{2\times \left(precision\times recall\right)}{precision+recall}$$

where TP, TN, FP, and FN represent the numbers of true positives, true negatives, false positives, and false negatives, respectively.

### 2.6. Analysis of the Differences in SNPs Selected by Various Methods

To visually understand the differences among the SNP selection methods of ANOVA, F-SCORE, and MIC, we employed the R package RIdeogram to map the distribution of the top 300 SNPs on rice chromosomes as identified by each method. Additionally, for a clearer comparison of the outcomes produced by different methods, we utilized the Python package Venn to generate Venn diagrams for the top 300 and top 1000 SNPs selected by each method.

## 3. Results

### 3.1. Suitable for Screening for Quantitative Traits

Based on the IFS strategy, the predictive accuracy results of three feature selection methods and four machine learning algorithms on the training set are summarized in Table 1 and Table S1. The results indicate that the KNN algorithm, in concert with different feature selection methods, has accuracy rates of 73.70%, 69.14%, and 70.82%. Overall performance is good, and the prediction accuracy is the highest in the framework when paired with the ANOVA feature selection method, achieving an accuracy rate of 73.70% while utilizing only 1170 SNPs. In addition, when paired with the ANOVA feature selection method, the RF algorithm demonstrated performance similar to that of KNN, which indicates to some extent that the core SNPs can be identified to be closely related to rice traits based on the PlantMine Framework and that ANOVA was effective in identifying core SNPs and eliminating redundant information. It is noteworthy that in the course of the training process, the Random Forest algorithm, in conjunction with ANOVA, FS, and MIC feature selection methodologies, demonstrated exceptionally high accuracies (97.87%, 97.86%, and 97.88%, respectively). Nevertheless, the quantities of SNPs employed were considerably elevated (7190, 3360, and 3690, respectively), suggesting that the RF algorithm contributed to a certain extent of overfitting.

**Figure 2:**
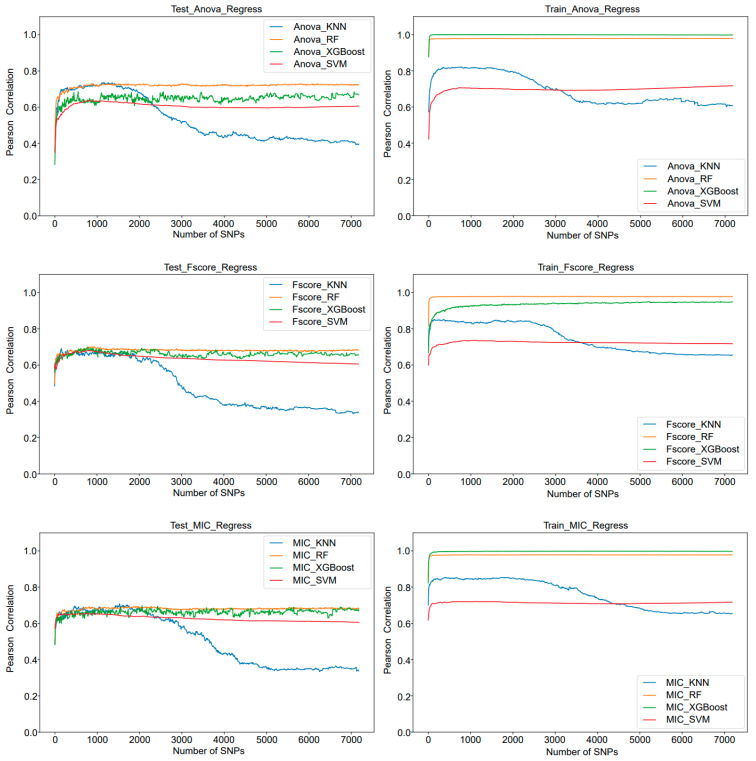
The IFS curves show the performance of three feature selections (ANOVA, F-SCORE and MIC) and the four models in the regress task. The blue curve represents KNN, yellow represents RF, green represents XGBoost, and red represents SVM.

In addition, as can be seen from Figure 2, under the IFS strategy, the prediction accuracy of each model reaches the maximum value with the increase in SNPs and gradually tends to be stable. However, the accuracy of the KNN algorithm decreases significantly when the number of SNPs required reaches a certain number under different feature selection methods, in which the decreasing trend is slowed down compared with the ANOVA method when paired with the F-SCORE and MIC methods. This implies that for quantitative traits, it is feasible to remove the redundant SNP information through appropriate feature selection, and the fluctuation of its accuracy may be due to the fact that the KNN algorithm is a distance metric-based method, and in the high-dimensional space, the distances between the sample points become sparse, which leads to the failure of the distance metric and thus the accuracy of the KNN algorithm is affected. Considering that ensuring performance while minimizing the number of SNPs is beneficial for reducing subsequent computational difficulties, the ANOVA as a feature selection strategy, performed particularly well. These findings provide strong support for the efficient identification and utilization of core SNPs in rice breeding, showcasing the immense potential of combining feature selection with machine learning algorithms in the analysis of genomic data.

### 3.2. Suitable for Screening for Quality Traits

**Figure 3:**
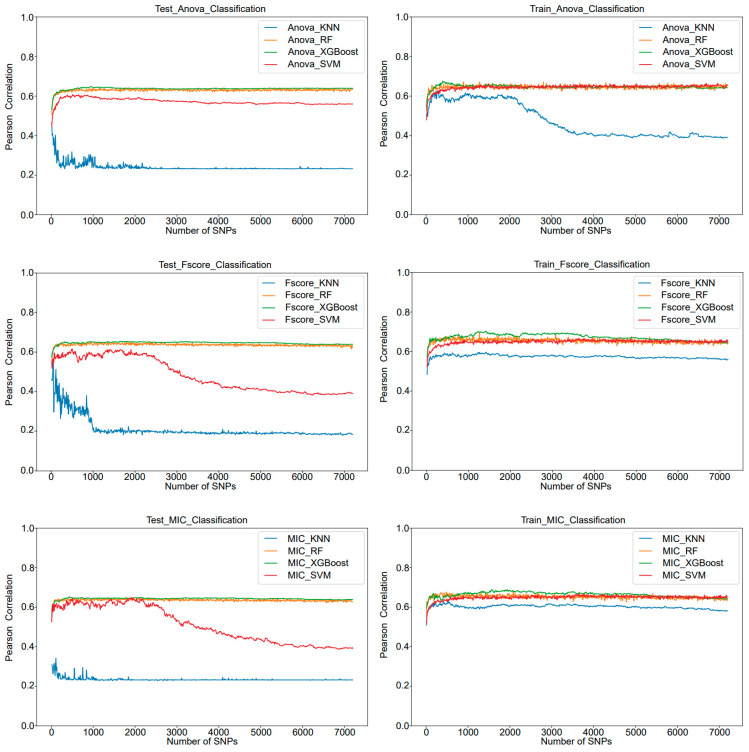
The IFS curves show the performance of three feature selections (ANOVA, F-SCORE, and MIC) and the four models in the classification task. The blue curve represents KNN, yellow represents RF, green represents XGBoost, and red represents SVM.

The approach of integrating feature selection with machine learning is equally applicable to the classification tasks of qualitative traits. As can be seen in Table 2 and Table S2, within the test dataset, the overall performance of the SVM model is excellent, and the SVM model utilizing F-SCORE shows the optimal performance, achieving an accuracy of 65.48%. The second good prediction performance is the SVM model utilizing MIC, which requires much fewer SNPs than under F-SCORE methods, but the accuracy is only 0.17% lower than F-SCORE, respectively. Furthermore, as depicted in Figure 3, aside from the KNN algorithm, which demonstrates a trend of performance decline with an increase in the number of features, indicating instability, the MIC feature selection method is capable of achieving comparable predictive performance to F-SCORE across different models with fewer features. This underscores the superior generalization ability and optimal overall classification performance of the MIC method in this classification task.

### 3.3. The Differences in SNPs Selected by Various Methods

**Figure 4:**
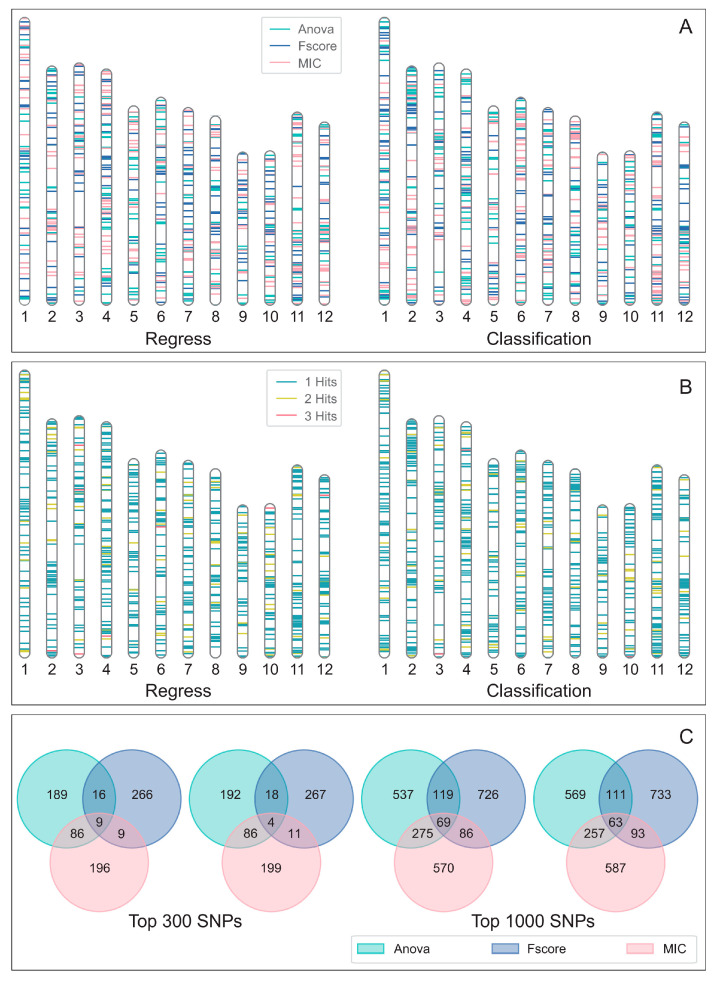
Differences between various feature selection methods: (**A**). Distribution of the top 300 SNPs selected by different feature selection methods on rice chromosomes (left is regression tasks, right is classification tasks). Green represents ANOVA, blue represents F-SCORE, and red represents MIC. (**B**). Intersection of results from different feature selection methods (left is regression tasks, right is classification tasks). Also using the top 300 SNPs, blue represents SNPs selected by only one method, yellow represents SNPs selected by two methods, and red represents SNPs selected by all three methods. (**C**). Venn diagrams of features selected by different methods. From left to right: regression tasks with the top 300 SNPs, classification tasks with the top 300 SNPs, regression tasks with the top 1000 SNPs, and classification tasks with the top 300 SNPs.

The top 300 SNPs selected by different methods exhibit distinct tendencies (Figure 4A, Supplementary S3), regardless of whether the task is regression or classification. Among the three methods, the important SNPs selected by MIC are notably more dispersed across the chromosomes, while F-SCORE tends to select SNPs close to key regions, and the results of ANOVA between these two approaches. This variation is clearly visible on chromosome 12, where the blue representing F-SCORE is concentrated in certain areas, while the red representing MIC displays a different trend. Figure 4B shows the varying degrees of overlap in the selection results of the three methods for both regression and classification tasks. It is noteworthy that only a very small number of SNPs are selected by all three methods. Significantly, SNPs at the end of chromosome 3 are selected by all three methods in both regression and classification tasks, suggesting that this region may play a crucial role in the development of the target phenotype. Figure 4C further illustrates the intersections of the top 300 and top 1000 SNPs selected by different methods. Within the top 300 SNPs chosen by each method, only a very small fraction (9 in regression, 4 in classification) are selected by all three methods (Table S3). When the scope is expanded to the top 1000 SNPs, this proportion increases (69 in regression, 63 in classification).

## 4. Discussion

Although machine learning has increasingly become an important research tool in botanical fields such as genomic prediction, feature selection is important to ensure the quality of data input for machine learning algorithms [26]. In prediction tasks, challenges such as information redundancy and noise are common; not every feature of high-dimensional data is effective for model prediction; irrelevant features reduce the accuracy of the algorithm; and for larger datasets, a higher search space not only increases the computation time but also affects the model’s generalization ability [26,27].

We have developed a computational framework capable of effectively identifying core SNPs closely associated with rice traits, as illustrated in Figure 1. The essence of this study lies in the integration of feature selection with machine learning algorithms, a strategy based on training with genomic variation data aimed at identifying core SNPs sets related to specific traits. When dealing with high-dimensional feature data, challenges such as overfitting, information redundancy, and noise are common. These issues not only reduce the generalization ability of the model but also affect predictive performance in cross-validation. Conversely, while low-dimensional features can enhance the robustness of the model, the limited number of features may not provide sufficient information, thereby impacting predictive accuracy.

To address these challenges and effectively manage high-dimensional data, selecting representative core SNPs is particularly crucial. This not only allows for a deeper understanding of the intrinsic properties of the rice genome but also improves the interpretability and accuracy of the predictive models. This study employs four machine learning algorithms and three feature selection methods, based on an IFS strategy, to efficiently mine the core SNP set related to rice breeding. The aim is to provide more precise genetic information for rice breeding, thereby facilitating the improvement of rice varieties and ensuring food security.

It merits emphasis that, based on our investigation, the efficacy of identical feature selection methodologies exhibits distinct variability contingent upon the nature of the task and the selection of computational models. This is particularly evident when dealing with quantitative traits, where the results are subject to disturbances from multiple factors. This may be due to regression tasks being highly sensitive, as they depend on the combined effects of numerous low-efficiency SNP loci. This further underscores the significance of the Plantmine framework, which acknowledges that no single feature selection method can universally apply to all problems and models. Instead, by integrating multiple conditions, it determines the most suitable methods and parameters for the intended task, ensuring a more tailored and effective approach.

For the same task, different feature selection methods might identify almost entirely different SNPs, as demonstrated in Figure 4, because each method analyzes data discrepancies from unique perspectives. For example, the MIC method focuses on detecting nonlinear correlations between variables, which allows it to capture minor effect loci dispersed throughout various regions of the genome. The substantial differences in how these algorithms perform can affect the results of genomic predictions when they are combined with different genomic prediction algorithms. Therefore, there is no single best feature prediction method but rather an optimal combination of feature selection and genomic prediction methods. This is why PlantMine provides a framework that includes feature selection, genomic prediction, and IFS curves instead of simply suggesting the best SNP selection method. As technology progresses and more rice datasets are developed, new feature selection methods and genomic prediction algorithms will emerge. When breeders are unsure about which feature selection algorithm to use or how to select core SNPs for genomic prediction, the technical framework offered by PlantMine can provide them with substantial guidance.

Furthermore, our research has uncovered several findings of significant referential value. Firstly, the performance of the KNN algorithm rapidly declines after reaching a peak as the number of features increases. This suggests to breeders that when directly predicting with high-density SNP datasets, the KNN model should ideally be avoided, whereas it might represent a viable option when fewer markers are employed. Secondly, simple feature extraction algorithms, such as ANOVA, can exhibit commendable performance in regression tasks. This perhaps elucidates why ANOVA remains the analytical tool most frequently selected in contexts such as field experiments; simplicity does not necessarily equate to inadequacy. Lastly, the stable performance of MIC in classification tasks underscores its formidable capability for extracting pertinent information.

## 5. Conclusions

This study presents a comprehensive computational framework designed to identify core SNPs that are closely associated with rice traits. By integrating feature selection techniques with machine learning algorithms, we have addressed the challenges posed by high-dimensional genomic data, such as overfitting, information redundancy, and noise, which often compromise model generalization and predictive performance. Our approach, which leverages an IFS strategy, has demonstrated significant efficacy in mining core SNP sets pertinent to rice breeding, thereby offering a pathway to enhance the precision of genetic information used in rice variety improvement and contributing to global food security efforts.

## Figures and Tables

**Table 1:** Comparison of accuracies of computational framework regression task.

Feature Selection Methods	Machine Learning Algorithms	Optimal Features	Accuracy (%)
ANOVA	KNN	1170	0.737001
	SVM	790	0.636433
	XGBoost	510	0.607868
	RF	880	0.730588
F-SCORE	KNN	170	0.691149
	SVM	800	0.6801
	XGBoost	790	0.698819
	RF	950	0.701213
MIC	KNN	1540	0.70826
	SVM	740	0.655904
	XGBoost	2070	0.698285
	RF	2290	0.694212

**Table 2:** Comparison of accuracies of computational framework classification task.

Feature Selection Methods	Machine Learning Algorithms	Optimal Features	Accuracy (%)
ANOVA	KNN	10	0.466071
	SVM	940	0.647621
	XGBoost	510	0.607868
	RF	5210	0.642267
F-SCORE	KNN	50	0.546494
	SVM	1660	0.654765
	XGBoost	500	0.616071
	RF	1170	0.65253
MIC	KNN	110	0.342857
	SVM	430	0.652978
	XGBoost	500	0.65
	RF	1790	0.650299

## Data Availability

Data will be made available on request. The ranking results for the importance of SNPs by three different methods are provided in Supplementary S1. We declare that the data generated in this work are based on our recognition of the spirit of the Toronto Statement. These data are merely a reordering of the SNP data provided by the 3 K Rice Genomes, using our method. These data are solely used for validating our framework. The raw dataset is available at the SNP-Seek database (http://snp-seek.irri.org/download.zul, accessed on 15 December 2023).

## Supplementary Materials

The following supporting information can be downloaded at: https://www.mdpi.com/article/10.3390/genes15050603/s1, Table S1: Performance of different combinations on the test and training datasets in regression tasks. Table S2: Performance of different combinations on the test and training datasets in Classification tasks. Table S3: The intersection of the top 300 SNPs selected by different feature selection methods. Figure S1: Schematic of the method for generating classification labels. Supplementary S1: The results of ranking SNPs by importance using different feature selection methods. Supplementary S2: The raw regression labels and the classification labels. Supplementary S3: The top 300 SNPs selected by different feature selection methods.
