# Supplementary material for: PlantMine: A Machine-Learning Framework to Detect Core SNPs in Rice Genomics
Source: Genes (Basel). 2024 May 9;15(5):603. doi: 10.3390/genes15050603 (PMC11120712; doi:10.3390/genes15050603)
Supplement: Supplementary file 1 [file genes-15-00603-s001.zip › Tables S1-S3.pdf]

Table S1. Performance of different combinations on the test and training datasets in regression tasks

Data set	Feature selection methods	Machine learning algorithms	Optimal features	Accuracy（%）
Train	anova	KNN	760	0.821003
		SVM	7150	0.717382
		XGBoost	770	0.999854
		RF	7190	0.978797
	fs	KNN	360	0.851882
		SVM	1080	0.735577
		XGBoost	6910	0.950806
		RF	3360	0.978604
	mic	KNN	1750	0.854016
		SVM	840	0.720951
		XGBoost	4100	0.998962
		RF	3690	0.978831
Test	anova	KNN	1170	0.737001
		SVM	790	0.636433
		XGBoost	510	0.607868
		RF	880	0.730588
	fs	KNN	170	0.691149
		SVM	800	0.6801
		XGBoost	790	0.698819
		RF	950	0.701213
	mic	KNN	1540	0.70826
		SVM	740	0.655904
		XGBoost	2070	0.698285
		RF	2290	0.694212

Table S2. Performance of different combinations on the test and training datasets in Classification tasks

Data set	Feature selection methods	Machine learning algorithms	Optimal features	Accuracy（%）
Train	anova	KNN	260	0.625
		SVM	390	0.675
		XGBoost	6960	0.664149
		RF	890	0.671429
	fs	KNN	1260	0.595811
		SVM	1420	0.703571
		XGBoost	3620	0.666374
		RF	1260	0.692857
	mic	KNN	520	0.62885
		SVM	1570	0.689286
		XGBoost	3620	0.666374
		RF	2080	0.682143
Test	anova	KNN	10	0.466071
		SVM	940	0.647621
		XGBoost	510	0.607868
		RF	5210	0.642267
	fs	KNN	50	0.546494
		SVM	1660	0.654765
		XGBoost	500	0.616071
		RF	1170	0.65253
	mic	KNN	110	0.342857
		SVM	430	0.652978
		XGBoost	500	0.65
		RF	1790	0.650299

Table S3. The intersection of the top 300 SNPs selected by different feature selection methods.

TASK	CHROM	POS	ID	ANNOTATION
Regress	2	29403	43300326	LOC_Os02g01040, LOC_Os02g01050, LOC_Os02g01060, LOC_Os02g01070
	3	35809832	115018005	LOC_Os03g63360, LOC_Os03g63370
	3	11141920	90350093	LOC_Os03g19800
	6	11557454	192640574	LOC_Os06g20140, LOC_Os06g20150
	10	680411	294165681	LOC_Os10g02070, LOC_Os10g02080
	3	4441485	83649658	LOC_Os03g08610, LOC_Os03g08620, LOC_Os03g08624, LC_Os03g08630
	12	3066511	348780174	LOC_Os12g06380, LOC_Os12g06400, LOC_Os12g06410
	4	32212743	147834735	LOC_Os04g54050, LOC_Os04g54060
	2	34844962	78115885	LOC_Os02g56850
Classification	4	4278367	119900359	LOC_Os04g08034
	10	23000354	316485624	LOC_Os10g42650
	3	35809832	115018005	LOC_Os03g63360, LOC_Os03g63370
	9	22932040	293404590	LOC_Os09g39980, LOC_Os09g40000

ANNOTATION column in the table: For each SNP, a 5kb region upstream and downstream, totaling 10kb, is analyzed to annotate the genes involved within this range.
