# Supplementary figures and images for: PlantMine: A Machine-Learning Framework to Detect Core SNPs in Rice Genomics
Source: Genes (Basel). 2024 May 9;15(5):603. doi: 10.3390/genes15050603 (PMC11120712; doi:10.3390/genes15050603)

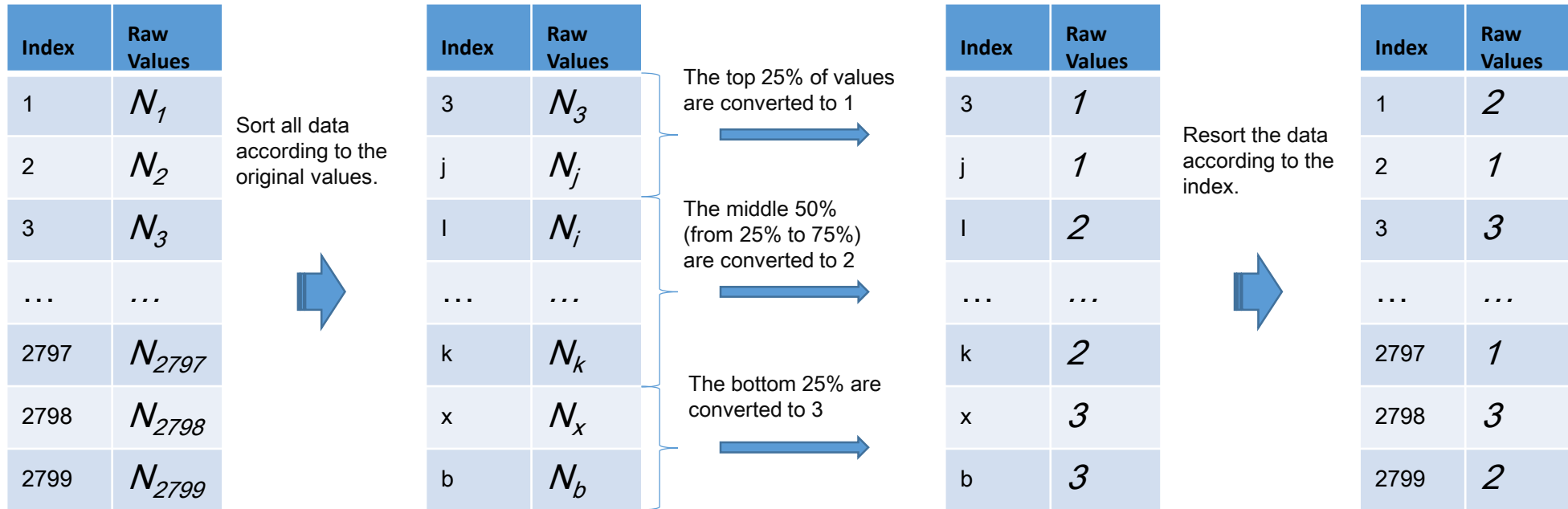

Figure S1. Schematic of the method for generating classification labels.

Supplement: Supplementary file 1 [file genes-15-00603-s001.zip › Figure S1.pdf]
